# RETRACTION

**DOI:** 10.3892/ijmm.2014.1990

**Published:** 2014-11-05

**Adseverin knockdown inhibits osteoclastogenesis in RAW264.7 cells**

WENTING QI, YAN GAO, JUN TIAN and HONGWEI JIANG

Int J Mol Med 34: 1483–1491, 2014; DOI: 10.3892/ijmm.2014.1941

All the authors agree to the withdrawal of this manuscript. The retraction has been agreed to as the authors have admitted that the manuscript contains a substantial amount of material from the following study: Hassanpour S, Jiang H, Wang Y, Kuiper JW and Glogauer M: The actin binding protein adseverin regulates osteoclastogenesis. PLoS One 9: e109078, 2014. Thus, this cannot be considered as original work.

